# Peer-Led Surgical Safety Learning Among Medical Students Using a Novel Story-Based Approach

**DOI:** 10.7759/cureus.10242

**Published:** 2020-09-04

**Authors:** Omar J Baqal, Mohammed Soheib, Amal A Saadallah

**Affiliations:** 1 Medicine, Alfaisal University College of Medicine, Riyadh, SAU; 2 Research Center/Safety & Scientist Genetics, King Faisal Specialist Hospital and Research Center, Riyadh, SAU; 3 Clinical Pathology, Ain Shams University College of Medicine, Cairo, EGY; 4 School of Advanced Studies, University of Phoenix, Phoenix, USA

**Keywords:** patient safety, surgical safety, medical education, peer learning, quality improvement, medical student, story learning, surgery, surgical skills, curriculum

## Abstract

Introduction

Even though medical students are important for the future of the overall healthcare system, they are often overlooked as valuable participants in safeguarding patient safety. Moreover, surgical safety is a critical topic that deserves to be addressed thoroughly during medical education, as part of the broader topic of patient safety. To maximize students’ engagement and to enhance their interest in patient safety and healthcare quality, it is imperative to explore and innovate stirring and interactive methods of learning. Through this paper, we present a unique narrative novel story-based, peer-led surgical safety teaching session organized by medical student representatives of Middle East's first student-led patient safety initiative and attended by medical students from various academic years.

Methods

A 1-hour case-based interactive learning session on surgical safety was developed, based on the World Health Organization (WHO) patient safety curriculum for medical schools. The session was peer-led at Alfaisal University College of Medicine, Riyadh and participation was voluntary. Learning objectives included surgical safety checklist, human factors, complexity of healthcare, students’ critical role in safety and open disclosure. The session included a verbal “story-telling” segment, followed by a didactic segment where learning objectives were covered. Attendees were continuously engaged throughout the session with the help of verbal inquiries. Pre-test and post-test questionnaires were distributed to assess participants' knowledge, awareness and perceptions regarding surgical safety and other objectives covered in the session.

Results

A total of 75 students participated in the session, consisting of 57.3% females and 42.7% males. Responses to the pre-test and post-test were analyzed and compared. Most students who attended the session reported that it was of value to them - with more than 90% of students considering the session either valuable or highly valuable. After the session, more students (10.67%, p = 0.10) were correctly able to identify that the initial reaction in healthcare is often individual blame (Q.1). Additionally, more students (30.63%, p < 0.001) were able to correctly identify the implementation of the WHO surgical safety checklist as the major factor that has contributed to the reduction of errors in healthcare (Q.2). Students’ responses also indicated that after attending the session a higher number (16%, p = 0.01) correctly identified that most errors linked to surgery were potentially preventable (Q.3).

Conclusion

Students are inherently willing to learn and engage in interactive learning. It was encouraging to see medical students show interest in this important patient safety topic, which also encourages similar future peer-learning initiatives. As reported, narrative story-based peer-assisted learning is an effective way to engage medical students in the cause of patient safety and should be utilized to further their knowledge and awareness regarding critical healthcare safety areas such as surgical safety, medication safety and infection control and instill a sense of responsibility in these future physicians.

## Introduction

Patient safety is critical to healthcare and is generally defined as the absence of preventable harm to a patient during the process of healthcare delivery and reduction of risk of unnecessary harm associated with healthcare to an acceptable minimum. Despite great advancements in healthcare, patient safety remains a matter of great concern, as innumerable patients are harmed daily due to medical errors and poor healthcare quality. Preventable medical adverse events cause 210,000-400,000 deaths every year in the United States, making it the third largest cause of death [1]. It is estimated that globally as many as four in 10 patients are harmed in primary and outpatient health care.

Medical students are the future of the healthcare system but are often overlooked or neglected as valuable participants in ensuring patient safety, possibly due to the perception that they lack clinical knowledge and experience. They may be discouraged to speak up and may hesitate to communicate their concerns, fearing negative evaluation. A study found that 76% of medical students had witnessed an error, highlighting their role into detection. However, only 7% of these students reported the error through the electronic error reporting system [2], highlighting the lack of student participation in safety activities.

Medical students play a potentially powerful role in medical error prevention as observed through case examples [3]. Their youthful energy, curiosity and drive to continuously learn can prove to be a great resource for the promotion of patient safety. There is a strong need for involvement of medical students in patient safety by engaging them in active training through knowledge and skills development. Medical students should be trained to recognize and to effectively report adverse events and medical errors. Such training can encompass various core aspects of patient safety including surgical safety, medication safety and infection control, and can be achieved through didactic lectures, hands-on workshops, awareness campaigns and use of technology such as social media.

Narrative learning

The usage of stories in medical education, research and practice, formally known as “narrative medicine” has well-supported benefits. Narrative learning is a powerful learning tool that can be utilized in several pertinent areas of training, such as meaning-making [4], development of identity [5], enhancing memory [5-7] and promoting empathy [8, 9]. It encourages trainees to view a patient beyond their clinical presentation and pay attention to their experiences and life events as human beings. Stories have been thought to be an effective way to promote understanding and embed new ideas [10]. Studies have highlighted the use of narrative-learning in medicine, noting that stories are frequently and intuitively used by physicians, assisting in teaching and building illness scripts [11, 12].

Peer-assisted learning

At the core of peer-assisted learning, or peer learning is the understanding that peer-teachers and their students share a similar knowledge base and learning experience, also known as “cognitive congruence”. This allows peer-teachers to use language and teaching modalities that their student-peers understand best, making learning more relatable. Besides a “cognitive congruence”, peer-teachers and students share a “social congruence” due to their similar social roles [13]. Thus, students feel more comfortable to engage in discussion and share ideas with a peer-teacher than with a senior clinician [14]. A systematic review highlighted several qualitative benefits of peer-learning strategies, including enhanced cognitive and psychomotor development of students as well as economic advantages [15].

There are several examples of attempts to formalize peer-learning on undergraduate and postgraduate levels. Peer-learning is particularly admired among medical students, giving them a platform to tackle challenging learning objectives [16,17]. Senior medical students tutoring their juniors are more likely to advise on useful revision strategies that are relatable to their student-peers. A USA study demonstrated that 99 of 130 respondent medical schools (76%) provided their students with some form of peer-learning during their medical program, while 57 (44%) also offered their peer-teachers formal training to support them in their teaching roles [18].

Surgical safety teaching in a narrative peer-assisted context

Surgical errors are of great concern owing to the serious negative impact they can have on patient health and safety. Complications after inpatient operations occur in about 25% of the patients, and at least half of the cases in which surgery led to harm are considered preventable. This has led to calls for surgical safety teaching to be introduced at an early stage in medical training. Information about the formalization of surgical safety teaching by medical institutions remains minimal, although several peer-assisted surgical skills teaching examples have emerged, including those among medical students and nurses [19, 20].

Through this paper, we share a unique teaching experience that interlinks all aspects mentioned above - 1) a narrative, 2) story-based, 3) peer-assisted (in fact peer-led), 4) surgical safety teaching experience. A one-hour interactive session was held at Alfaisal University College of Medicine, Riyadh, Saudi Arabia and was attended by medical students voluntarily. This session was designed, organized and delivered by student members of the Quality & Patient Safety Program (QPS) at Alfaisal University, with the support of a faculty scientific advisor. Established in 2012, to the best of our knowledge, QPS was then the first student-led patient safety initiative from the Middle Eastern region. It aims to engage students, other healthcare professionals and the general public through three main ways: awareness, education and research. The QPS program when first formed also facilitated then the establishment of the Institute of Healthcare Improvement (IHI) School Chapter at Alfaisal University.

## Materials and methods

The session was attended by 75 medical students, of which 57.3% were females and 42.7% were males. Fifty-two of the 75 attendees were first year medical students, while 13, six and four attendees were second, third- and fourth-year medical students, respectively. The initiative consisted of a 1-hour session that was developed based on the World Health Organization (WHO) Patient Safety Curriculum Guide for Medical Schools [21]. It was initiated and implemented by a group of medical students who were members of the Quality & Patient Safety Program (QPS) at Alfaisal University (AU), Riyadh, and were supervised and guided by a faculty scientific advisor. The session was named “Jameela's Misery: A QPS Case File”. Institutional approval was acquired from the AU College of Medicine (IRB-20058), including all aspects of organization and promotion of the session and sharing our experience scientifically.

The surgical safety case was inspired by Elaine Bromiley's tragedy [22] and was adapted to match local patient safety trends. In our story, the patient Jameela undergoes a gynecological surgery, an experience that is riddled with disasters, starting from the first appointment with the physician to events in the Operating Room during her surgery. The session was delivered with the help of a PowerPoint presentation and included verbal narration of a story, followed by a didactic section to cover learning objectives. Pre-test and an identical post-test were distributed to all attendees to assess participant knowledge, perceptions and attitudes towards surgical safety and students’ role in patient safety. The session was announced through two modes: email and the medical student association's social media platforms. Participation was voluntary and all AU medical students were eligible to participate.

The pre-test was distributed before (prior to beginning) the session upon the attendees' entrance into the lecture hall. Providing demographic details was optional but encouraged (Name, Student ID, Academic year). The test had 11 questions including three 5-point Likert scale questions (1-strongly disagree and 5-strongly agree), three multiple-choice questions (MCQs) in SBA (Single best answer) format and five Yes/No format questions. The test assesses students' attitudes, knowledge and intent to engage in patient safety.

An identical post-test was distributed after (upon completion) the session and before the students left the lecture hall to evaluate the immediate impact of the session on attitudes and knowledge, with minimal necessary modifications. The tests were reviewed for content validity by several faculty members with training in patient safety.

Session development

The learning objectives covered in this session, as adapted from the WHO Patient Safety Curriculum Guide for Medical Schools [21] listed below:

· The basics of surgical safety

· WHO’s Surgical Safety Checklist

· Understanding systems and impact of complexity on patient care

· Human factors

· Being a team player and showing leadership

· Managing fatigue and stress

· Involving patients and carers as partners in healthcare

· Communicating honestly with patients after an adverse event (open disclosure)

Details of the full story can be found in the Appendix. To encourage students’ interaction, the following questions were asked at various points during the verbal narration of the story (marked by * in the Appendix)

1. Can you identify any patient safety hazards so far? What could be done better?

2. What went wrong? What could have been done better?

3. What are your thoughts? How could all this have been prevented? What actions would you take?

Upon completion of the story, students were provided with certain scientific information to supplement learning and achieve the learning objectives. Such information was as follows. The complexity of healthcare was discussed and how that can increase the chances of producing adverse events. It was emphasized that medical error is most often not an individual failing, but systemic problems that make it easier to make mistakes. Reason’s “Swiss cheese” model of accident causation was used to add emphasis. Next, the characteristics of HROs (high-reliability organizations) were discussed, and the lessons healthcare can learn from other HROs, especially the aviation industry.

Human factors were defined and their importance in healthcare discussed, with the help of few examples (e.g., look-alike and sound-alike medications, equipment design). The impact of stress and fatigue on work performance was also elaborated. Interactive activities including optical illusions (are the lines straight or crooked, say the color of the word not the displayed word itself) were used to lift the mood and at the same time deliver an important message: to err is human.

Basic surgical safety concepts were introduced to the students, including main types of adverse events associated with invasive procedural and surgical care and ways to improve surgical safety, with a major emphasis on the WHO Surgical Safety Checklist [23]. The checklist was then correlated with the delivered patient story, to show how this disaster could have been prevented if the WHO Surgical Safety Checklist was used.

The importance of effective leadership in healthcare was discussed while highlighting the qualities of effective leaders. Participants were informed about the technique of successfully gaining informed consent and the key principles of open disclosure, which is informing patients and their families of bad outcomes of medical treatment, as distinguished from bad outcomes that are expected from the disease or injury being treated.

## Results

The questionnaire (see Tables 1-3) was completed by the students before and after the session. A total of 75 students had completed the questionnaire. A Shapiro-Wilk test was used to assess normality, and it was determined that the data was not normally distributed, therefore a Wilcoxon Signed-Rank test was used for non-parametric analysis of the Likert data. Cronbach’s alpha was used to determine the internal reliability (α = 0.70). McNemar’s test was used for the analysis of the dichotomous variables.

**Table 1:** Students’ attitudes

Attitude Items	Pre-test, mean (SD)	Post-test, mean (SD)	Difference pretest - posttest	p-value	Wilcoxon test-statistic
Q.1) “Patients and their families have the right to know about their treatment options, and about a mistake made during treatment, even though it may not have caused any harm.”	4.13 (.78)	4.75 (0.60)	0.62	.001	52.00
Q.2) “If we keep in mind the extent of human capabilities while designing work processes, we can reduce mistakes.”	4.09 (0.66)	4.76 (0.54)	0.67	.001	64.00
Q.3) How valuable do you perceive patient safety initiatives sessions to be?	4.20 (0.77)	4.71 (0.49)	0.51	.001	97.50

**Table 2:** Students' knowledge

Knowledge Items	Pre-test, % correct	Post-test, % correct	Difference %	p-value
Q.1) Which one is correct about errors in healthcare? Correct = In healthcare, the first reaction is to blame the individual when an error occurs.	60.00	70.70	10.70	.10
Q.2) What has WHO (World Health Organization) established to cause a great reduction in medical errors, specifically those surgical in origin? Correct = WHO surgical safety checklist tool.	50.70	81.30	30.60	.001
Q.3) Which of the following is not correct about errors in surgeries? Correct = Major errors during surgeries are non-preventable.	60.00	76.00	16.00	.01

**Table 3:** Measuring student intent to get involved in patient safety

Intention Items (Y/N) “I plan to… ”	Pre-test, % Yes	Post-test, % Yes	Difference %	p-value
Q.1) “Voice my opinions during my clinical training at the hospital.”	32.00	42.70	10.70	.07
Q.2) “Join a Quality & Patient Safety group.”	41.30	53.30	12.00	.08
Q.3) “Participate in patient safety research projects.”	38.70	41.30	2.60	.68
Q.4) “Report any safety hazards through the SRS (Safety Reporting System) at my training hospital.”	58.7	74.7	16.00	.014
Q.5) “Come up with ideas for healthcare performance improvement.”	46.7	58.7	12.00	.117

The questionnaire included Likert items that assessed students’ perceptions (Table 1). There was a positive increase in responses for items assessing the perception of students on patient-education and the appropriate disclosure of medical errors to patients (Table 1, Q.1) (W = 52, p < 0.05). Students were also able to recognize the impact of redesigning workflows in healthcare to build work environments conducive to minimizing errors (Table 1, Q.2) (W = 64, p < 0.05). Lastly, students' perceived-value in patient safety initiatives by the student-led groups also showed improvement (Table 1, Q.3) (W = 97.5, p < 0.05).

Overall most students who had attended the session reported that it was of value to them - with >90% of students considering the session either valuable or highly valuable. There was an improvement in all answers scores of the questionnaire (Table 2). After the session, more students (10.67%, p = 0.10) were correctly able to identify that the initial reaction in healthcare is often individual blame (Table 2, Q.1). More students (30.63%, p < 0.001) were able to correctly identify the implementation of the WHO surgical safety checklist as the major factor that has contributed to the reduction of errors in healthcare (Table 2, Q.2). Student responses also indicated that after attending the session a higher number of students (16%, p = 0.01) correctly identified that major errors during surgery were potentially preventable (Table 2, Q.3).

The questionnaire also included five dichotomous questions that assessed the students’ beliefs about their role in the healthcare environment as medical students (Table 3). The students were asked about their willingness to voice their opinion on patient safety issues during their time at the hospital (Table 3, Q.1). After the students had attended the session, there was a 10% increase (p = 0.07) in the students’ positive responses to the prompt. Students were also asked if, they as medical students, believe they have a role in reporting hazards to the Safety Reporting System (Table 3, Q.4). After attending the session more students responded affirmatively (16%, p = 0.014).

The students’ willingness to involve themselves in the local patient safety advocacy group (Table 3, Q.2) (difference = 12%, p = 0.08) and patient safety research projects (Table 3, Q.3) (difference = 2.60%, p = 0.68) was assessed by two questions which in the post-session test showed increases in the positive responses - although these did not reach statistical significance. Lastly, after completing the session, more students believed that they, as medical students, have a role in improving the safety of patients by coming up with creative solutions for healthcare improvement projects (Table 3, Q.5) (difference = 12%, p = 0.117).

## Discussion

To the best of our knowledge, this is the first narrative story-based, peer-led surgical safety learning intervention at a medical school level in the Middle Eastern region, if not the whole world. Given the central importance of surgical safety in modern patient safety and quality improvement research and education, we intended to make the WHO Surgical Safety Checklist an important focus of our session. Additionally, to inspire and motivate students to engage further in the cause of healthcare safety and quality improvement, we aimed to anchor the concept that a single initiative as simple as a checklist, can make a considerable impact on patient safety. Overall, we found improved awareness about the role of the WHO Surgical Safety Checklist in patient safety and how the students can participate in promoting and ensuring surgical safety. More than 90% of participants found the session valuable, reflecting the potential benefit of such a learning approach.

Recognizing the value of narrative learning, a previously published study shared a theoretical structure for the use of story in higher education, while providing illustrative examples from several academic fields, including nursing [24]. Besides medical students, peer-learning has also been explored among other academic trainees, such as nursing and physiotherapy students [25,26]. In addition to the wide range of benefits peer-learning brings to the learners, it can also prove valuable for institutions, particularly those with limited availability of teaching staff. Such institutions are recommended to facilitate peer-learning, by providing their students with necessary support and resources to enhance its effectiveness and utility as a learning approach.

In light of evidence highlighting that many medical students lack basic surgical safety skills and knowledge [27], the authors believe that no medical student should graduate without core knowledge of patient safety, including surgical safety. A national UK cross-sectional study on medical and nursing students found that UK undergraduate surgical safety checklist training did not meet the minimum standards set by the WHO [28]. Several research studies are supporting the use of a wide range of teaching methods to encourage deep learning [29,30], with WHO recommending the usage of experimental learning for critical thinking [21]. Usage of interactive and multi-media teaching methods was central to our approach, aiming towards eliciting intense feelings from the attendees towards the surgical tragedy in our story, through questions and imagery.

For future directions, we would recommend exploration of this teaching approach in clinical clerkship medical students as well, who undertake rotations at the hospital. Based on local patient safety issues and interests, topics could be chosen to supplement the students' medical education. The sessions could be delivered as stand-alone modules on different days or as a series on the same day. It is imperative to provide each topic sufficient time for story delivery, student engagement and optional assessment. We found 1 hour to be sufficient, but we would have needed more time should a practical and hands-on teaching element be incorporated into the session.

In light of the COVID-19 pandemic, as more and more medical students and trainees are responding to the call of duty, it would be interesting to see this teaching style be implemented for COVID-19-related topics such as infection prevention and control, critical care skills, etc. Through our paper, we have referenced over 10 studies sharing their experience with story-based learning, peer-learning and peer-led surgical skills learning among medical trainees that provide valuable background information to readers.

The fewer number of 4th and 5th year student attendees could be due to the fact that these students undertake clinical clerkships at affiliated hospitals and were not present at the university campus during the session. Despite a few apparent limitations - it being a 1-hour one-time session and attended by a relatively modest group of around 75 students - the positive feedback reinforces the potential of this approach and the possibility to expand to other important patient safety topics such as infection control and medication safety in the near future.

## Conclusions

It is imperative that all medical students graduate with a basic understanding of surgical safety. This can be achieved by the utilization of newer interactive methods of teaching to improve students’ interest and participation. Peer-assisted learning consolidated by the power of stories has the potential to enhance the development of medical students into safe and responsible physicians, as well as the patient safety ambassadors of the future.

We were encouraged by the student turnout, interest shown and very positive feedback from the attendees, giving us the motivation to undertake further peer-led patient safety peer-learning projects going forward. This is a step towards ensuring that patient safety emerges and remains at the forefront of the rapidly evolving medical education curriculum, with the learners being the drivers of this transformation.

## Appendices

Full story:

(Note: To encourage student’s interaction, certain questions were asked at different points [(marked by * below], during the verbal narration of the story)

Mr Qasim’s wife, Jameela was a university lecturer. She had been suffering from long-standing abnormal uterine bleeding. After having consulted a gynecologist regarding her problem, she was eventually scheduled for a hysterectomy. They felt like they were not properly briefed about available treatment options and were made to take this decision in a rush. Jameela was 31 years old, and she always wished to have more children in the future, besides the 6-year-old daughter they had. Jameela was given some papers to sign but wasn’t given a chance to read through them. She was told it was the “consent form”. *

On the day of the surgery Jameela was clearly nervous as she was being taken to the operating room. The surgeon had just finished another hysterectomy after having completed a night shift. The surgical team was made up of 8 people, some of whom were working with the surgeon for the first time. The team didn’t anticipate any complications.

As Jameela was being put under general anesthesia, the anesthesiologist attempted intubation, but faced difficulty. This was an anesthesiologist with 16 years of experience. Suspecting tension around her jaw muscles, muscle relaxants were injected. By this time, Jameela’s oxygen saturation was dropping, now at 70%. She had begun turning cyanotic. The anesthesiologist from the neighboring theater came over to help out. Nurses tried their own methods too with different pieces of equipment, but they were told by the surgeon that they were “overreacting” and needed to “relax”. All intubation attempts had failed, oxygen saturation continued dropping, now to 50%. 20 minutes into the commotion, the team was finally able to intubate her and bring the oxygen saturation back to 90%. The anesthesiologist stepped out of the operation room to tell Mr Qasim about the difficulty they had faced intubating Jameela but reassured him that everything was under control. *

The surgery proceeded. Mid-way through the procedure, the team encountered profuse bleeding from the uterine artery. Jameela was losing a lot of blood. The surgeon tried several ways to control the bleeding but failed. Given the amount of blood Jameela had lost, the surgeon yelled for replacement blood packs. At that point, the team realized they just didn’t have enough blood available for replacement, as it hadn’t been arranged earlier. This was the point of no return. The surgeon stood over Jameela’s body helplessly, knowing very well that all this could have been prevented had they taken some simple safety precautions. *
